# Nucleotide supplementation: a randomised double-blind placebo controlled trial of IntestAidIB in people with Irritable Bowel Syndrome [ISRCTN67764449]

**DOI:** 10.1186/1475-2891-5-16

**Published:** 2006-06-08

**Authors:** CP Dancey, EA Attree, KF Brown

**Affiliations:** 1 University of East London, UK

## Abstract

**Background:**

Dietary nucleotide supplementation has been shown to have important effects on the growth and development of cells which have a rapid turnover such as those in the immune system and the gastrointestinal tract. Work with infants has shown that the incidence and duration of diarrhoea is lower when nucleotide supplementation is given, and animal work shows that villi height and crypt depth in the intestine is increased as a result of dietary nucleotides. Dietary nucleotides may be semi-essential under conditions of ill-health, poor diet or stress. Since people with Irritable Bowel Syndrome tend to fulfil these conditions, we tested the hypothesis that symptoms would be improved with dietary nucleotide supplementation.

**Methods:**

Thirty-seven people with a diagnosis of Irritable Bowel gave daily symptom severity ratings for abdominal pain, diarrhoea, urgency to have a bowel movement, incomplete feeling of evacuation after a bowel movement, bloating, flatulence and constipation for 28 days (baseline). They were then assigned to either placebo (56 days) followed by experimental (56 days) or the reverse. There was a four week washout period before crossover. During the placebo and experimental conditions participants took one 500 mg capsule three times a day; in the experimental condition the capsule contained the nutroceutical substances. Symptom severity ratings and psychological measures (anxiety, depression, illness intrusiveness and general health) were obtained and analysed by repeated measures ANOVAs.

**Results:**

Symptom severity for all symptoms (except constipation) were in the expected direction of baseline>placebo>experimental condition. Symptom improvement was in the range 4 – 6%. A feeling of incomplete evacuation and abdominal pain showed the most improvement. The differences between conditions for diarrhoea, bloating and flatulence were not significant at the p < .05 level. There were no significant differences between the conditions for any of the psychological measures.

**Conclusion:**

Dietary nucleotide supplementation improves some of the symptoms of irritable bowel above baseline and placebo level. As expected, placebo effects were high. Apart from abdominal pain and urgency to have a bowel movement, the improvements, while consistent, are modest, and were not accompanied by improvements in any of the psychological measures. We suggest that the percentage improvement over and above the placebo effect is a physiological effect of the nucleotide supplement on the gut. The mechanisms by which these effects might improve symptoms are discussed.

## Background

Irritable Bowel Syndrome (IBS) is a chronic disorder affecting an estimated 15 – 22% of western populations, and is the major cause of referrals to gastroenterology clinics in the western world [1]. The symptoms may include abdominal pain, diarrhoea, urgency to have a bowel movement, a feeling of incomplete evacuation after a bowel movement, flatulence and bloating. Some sufferers experience constipation, or an alternation between constipation and diarrhoea. IBS is more common among women than men, with a 2:1 female: male ratio [2]. Current medical treatments are directed at symptomatic relief, and although these can give some relief, there is no one treatment which has been shown to be lastingly effective. Although IBS is not a life-threatening disease, the symptoms and the effects of the symptoms on daily life can have a great impact on sufferers [3]. IBS is also associated with large healthcare and economic costs in terms of hospital investigations, repeated visits to GPs, prescription medicines, and loss of time from work [4]. Although hospital investigations for more serious diseases such as cancer or Inflammatory Bowel Disease are negative in people with IBS, some abnormalities in the gut have been found. For instance, some patients have been found to have a degree of mucosal inflammation, which may be in response to some foods [5]. It is possible that people with IBS have immunological reactions to dietary antigens as food elimination based on serum immunoglobulin G antibodies has been found to result in a significant decrease in symptoms of IBS. Both numbers of mast cells and their mediators have been shown to be increased in intestinal mucosa in patients with irritable bowel syndrome, especially in the close proximity of intestinal nerves [6]. Kalliomäki [6] suggests that food antigens induce mast cells to secrete mediators which regulate gastrointestinal motility, resulting in alterations in peristalsis and an increase in abdominal pain and discomfort. Furthermore, the mast cell-derived mediators have effects on immune cell functions. It may be then, that the nutrition of people with IBS is more important than has been traditionally thought. As people with IBS tend to believe that their symptoms are affected by diet, they often attempt to manage their disorder by dietary control. However, the only consistent advice given to people with IBS is usually simply to eat a "healthy" diet which includes fruit, vegetables and fibre. In an early study of people with IBS, Dancey & Backhouse [7] found that although the majority of their sample of 148 people (70%) stated that they were trying to follow a "healthy" diet with large amounts of fruit and vegetables; for many of these people, such a strategy had not led to symptom improvement, and in an attempt to control their IBS, 14% were eating very restricted diets. Some of these diets involved avoiding complete groups of foods, e.g. carbohydrates. Whilst such a strategy may reduce bloating, for instance, such a diet is not likely to enhance wellbeing. As well as eating a sufficient quantity of a wide variety of foods from each food group, micronutrients and nucleotides may also be important for health, especially in the sub-well. It is nucleotides which are the focus of this study.

Nucleotides are substances which are synthesised endogenously – they have important effects on the growth and development of cells which have a rapid turnover, such as those in the immune system and the gastrointestinal tract. The intestinal epithelium is a rapidly proliferating tissue with a high cellular turnover rate. A complete cell cycle in humans is 24 hours, with a replacement of the entire enteric epithelium within 3–6 days. In healthy people, dietary nucleotides are probably not essential, and in fact most will be metabolised and rapidly excreted from the system. However, under certain circumstances (e.g. in the sub-well, diseased, or under conditions of stress or poor diet) dietary nucleotides may be what Maldonado, Navorro, Narbona, & Gil [8] call "semi-essential", optimising the function of the gastrointestinal and immune systems. In relation to the gastrointestinal system work has shown that dietary nucleotides enhance the intestinal absorption of iron [9]. Dietary sources of nucleotides are nucleoproteins and nucleic acids, and these are found to varying degrees in many foods – lamb, liver, mushrooms (but not fruit and other vegetables) all are rich in nucleotides. Rapidly dividing tissue requires a constant supply of nucleotides in order to manufacture essential nucleic acids. Exogenous supplies of nucleotides may optimise tissue function particularly during recovery from mucosal injuries when the endogenous supply may limit the synthesis of nucleic acids.

Holen & Jonsson [10] found that dietary nucleotides had beneficial effects, especially when the nutrition supply was inadequate. Work with infants has shown that the incidence and duration of acute diarrhoea is lower in infants when dietary nucleotides are included in their diets [11]. Previous work on the effect of nucleotide supplementation in animals has found that such supplements are important for the repair mechanism of immune cells [12]. In piglets, nucleotide supplementation had effects on the gastrointestinal system by increasing villi height and crypt depth. [13]. Evans, Tian, Gu, Jones & Ziegler [14], using rats to model short-bowel syndrome, found that nucleotide supplementation is associated with increased jejunal adaptive growth after massive small bowel resection in rats. Dietary nucleotides have been found to help athletes by reducing the release of stress related hormones and chemicals in the body, and by maintaining a higher level of antibodies, which enables the immune system to work more effectively [15]. In people with a chronic illness such as IBS whose primary symptoms relate to the gastrointestinal tract, nucleotide supplementation may improve symptoms via improved gut function or by an enhancement of the immune system.

There are particular problems in assessing the benefits of treatments of people with IBS, and some of the problems of this patient group in relation to clinical trials have been discussed in detail by Spiller [16]. People with IBS show great variability in frequency and severity of symptoms, both when compared to others and also from day-to-day in their own symptoms. Spiller [16] has shown there are clear benefits to participating in clinical trials; people with IBS tend to be helped by placebo alone. This is thought to be due to a reduction in anxiety and/or depression as a result of help and reassurance given by the people running the trial. If this is the case, then anxiety and depression should reduce over the length of the trial. One would also expect that, if symptoms improve, then psychological well-being should in some way improve also. Thus although our primary aim was to determine whether nucleotide supplementation improved the symptoms of irritable bowel, we also wished to determine whether ratings of anxiety and/or depression would show any change as a result of symptom change.

## Methods

### Design

A double-blind randomised placebo cross-over study in which participants rated symptoms daily for six months in baseline, placebo, and experimental conditions. This study was approved by our university ethics committee and was in accordance with the code of conduct for psychologists (ethical principles for conducting research with human participants) produced by the British Psychological Society. The research was conducted only when written consent was received from the participant, and their G.P. had provided written confirmation of their diagnosis, and confirmation of no other co-existing conditions.

### Participants

**Figure 1:**
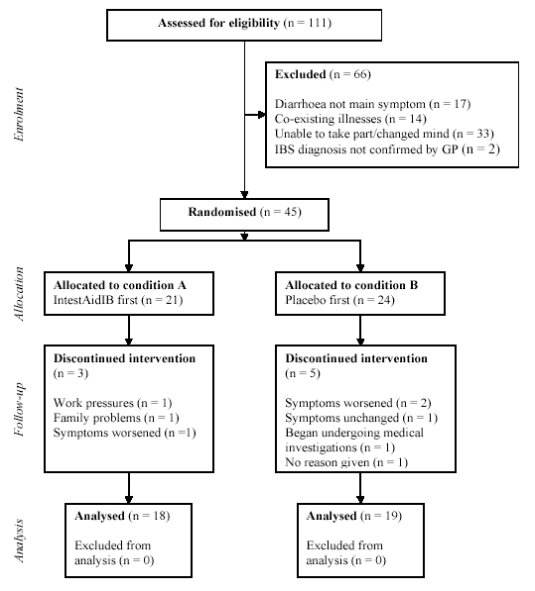
participant flow through the trial.

Thirty seven people with a diagnosis of Irritable Bowel Syndrome took part in the study. The inclusion criteria were that participants should be aged 18 – 65, should have been diagnosed as having IBS by a qualified medical practitioner, and should have diarrhoea as a main symptom. The exclusion criteria were any other co-existing illnesses, and non-confirmation of the diagnosis by G.P. The participant flow is shown below (see Figure 1).

Thirty-seven participants completed the trial, during which they completed daily symptom diaries for four weeks (baseline) following by either the experimental condition (eight weeks) or the placebo condition (eight weeks). There was a four week washout period between conditions. The mucosal cell turn-over for the whole tract is approximately six days, with the majority of other cells in the body are also replaced over 30 days. For this reason a 30 day washout was selected. Although some of the participants rated some symptoms as not particularly bothersome over the baseline condition no person was excluded by us.

### IBS symptoms

The symptoms of interest were: abdominal pain, diarrhoea, urgency to have a bowel movement, incomplete evacuation after a bowel movement, flatulence, bloating, and constipation. Participants rated symptoms every day on a scale of 1 ("no discomfort at all today") to 7 ("very severe discomfort today"). Reliability for these symptoms has been previously established [17].

Participants were given a pack containing a month's supply of daily diaries in which to record their symptoms. The next month's supply were sent to the participants once the previous completed pack had been returned.

### Neutroceutical product

Participants took one 500 mg capsule three times a day with meals – in the experimental condition these contained the neutroceutical product IntestAidIB which consists of the following active substances: nucleotides & RNA (concentrated extracts of Saccharomyces cerevisae), hydroxypropyl methycellulose, FOS (fructo-olligosaccharides), Methionine, Glutamine, Inositol, Lysine, Pantothenic acid (Vitamin B5 as Calcium d-pantothenate), Sodium citrate, Riboflavin (Vitamin B2), Vanillin, Folic acid, and Biotin. Flow agents are magnesium stearate and silicic acid. The capsules are carbohydrate based. RNA and the specific, purified nucleotides are the natural extracts of yeast. There are no yeast cells carried over from the extraction process into the product. The daily diaries contained space where participants confirmed that they had taken each capsule, and at what time of day.

### Procedure

Recruitment was by a variety of sources. Notices asking for people with diagnosed IBS interested in taking part in a trial for a neutroceutical product were sent to people with IBS who had previously expressed an interest in IBS-related research; a request was included in one issue of Gut Reaction (a quarterly journal sent to people with IBS by The IBS Network, a British self-help organisation), and notices were placed in doctors' surgeries and libraries. Such a recruitment strategy was considered necessary, as strict inclusion and exclusion criteria, plus the burden of completing daily diaries for six months meant recruitment might be difficult. We wished to have a mixed group of people with IBS, representative of the general population of people with IBS. Recruiting from tertiary centres does not allow generalisation to the wider population of people with this condition.

Participants were invited to respond to these recruitment methods by telephone or e-mail. During this initial contact, the researcher ensured that potential participants met the inclusion criteria. Eligible individuals were then given full information relating to the study. They were advised at this stage of their right to withdraw their participation and/or any data already provided, at any time, without giving any notice or reason to the research team.

Participants were then asked to provide contact details for their GP, and a consent form was sent by post to the participant for them to complete and return, as well as a pack providing further information about the trial and the supplement. Their GP was also sent a letter at this point, summarising the trial and requesting confirmation of diagnosis. Upon receipt of the confirmation of diagnosis and participant consent forms, participants were randomised to each of the conditions.

Initially participants were sent their first symptom diary pack, which contained detailed instructions on how to complete the diary as well as the first diary itself. The diary required participants to rate the severity of their symptoms (see above) and to specify the times at which they had taken each of their three capsules; to name any medications which they were prescribed, had purchased (including price) and had actually taken (including time taken) that day; and to note any visits to health professionals made that day (including who they visited, reason for visit and advice given).

Participants were contacted by telephone or e-mail a week after this first pack was sent out (week 1), to confirm that they understood how to complete the questionnaires and the diary. Participants were sent their first set of capsules (a "set" comprised four sealed tubs) – all tubs were marked "nutritional supplement, contents 42 capsules (500 mg), two weeks supply", but with A or B clearly marked both on the label and on the cap) one week before they were to need them (week 3), with a letter stating the date on which they should begin taking the capsules. They were contacted by telephone or e-mail a few days after this date (week 5) to ensure that they were taking the capsules at the right dosage (one capsules three times a day, preferably with meals), that they were completing the symptom diary with the required information (time capsules taken, any missed capsules noted), and that they were aware of how to contact the research team if they had concerns about any effect which the capsules may have upon them.

Following this, participants were sent a symptom diary pack every month, their second set of capsules for week 17, a set of questionnaires (described below) at week 13 and week 25. All documentation and capsules were sent one week before they were required to ensure that they arrived in good time. Every letter sent to the participants included contact details for the research team and urged participants to telephone or e-mail with any questions or worries, at any stage of the trial. Participants were sent questionnaire packs at baseline, washout, and end of trial.

Every participant forgot to take at least one capsule across the duration of the trial, but this was usually one capsule only on any given day. There were two exceptions – one participant took capsules erratically over the last six weeks of the experimental condition and after the first five weeks of the placebo condition. This participant had a change of personal circumstances during this time (she underwent a hysterectomy). The other participant took no capsules for 5 days in the last week of the experimental condition as she went away and forgot to take the capsules with her.

### Psychological measures

Measures were taken at the beginning of the baseline period, the end of the experimental condition and the end of the placebo condition.

Depression was measured by the CES-D [18] and anxiety was measured by the Stait-Trait Anxiety inventory [19]; a specific measure of health anxiety was provided by the Health Anxiety Questionnaire [20]. General health and happiness were measured by the total of the GHQ-60 [21] and the Affect Balance Scale [22]. The extent to which IBS intrudes into various aspects of everyday life was measured by the 13-item Illness Intrusiveness Rating Scale [23].

## Results

The average person with IBS spent approximately £15.00 during the six months trial on medications, supplements, minerals and vitamins and visited the G.P.'s surgery approximately seven times, the majority of the consultation times being for reasons other than IBS.

### Symptoms

For symptom recording, three participants had some sequential data missing, i.e. for the first participant this was for seven days in the experimental condition; for the second it was three days in the experimental condition, and for the third, three days during baseline. For each missing data point frequency data were obtained for the particular condition in which the missing data occurred, and the most appropriate (representative) measure of central tendency was inserted.

### Analysis of symptoms

**Figure 2:**
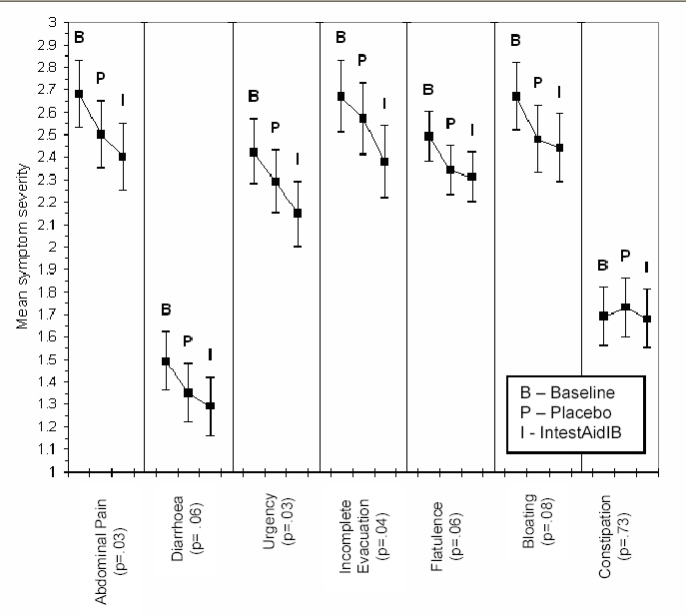
mean symptom severity ratings with 95% C.Is.

Mean ratings for the symptom series for each condition with 95% C.I.'s for the symptoms are represented below (see Figure 2).

A repeated measures ANOVA on each of the symptoms was carried out. Sphericity was not assumed and therefore the Greenhouse-Geisser correction for degrees of freedom was used. The difference between conditions for abdominal pain (F_2,67 _= 3.71; Eta^2 ^= .10), urgency to have a bowel movement (F_2,64 _= 3.82; Eta^2 ^= .10) and a feeling of incomplete evacuation (F = _2,67 _= 3.52; Eta^2 ^= .09) were significant at p < .05. Diarrhoea (F_2,58 _= 3.08; Eta^2 ^= .08), Flatulence (F_2,70 _= 2.89; Eta^2 ^= .07), Bloating (F_2,68 _= 2.61; Eta^2^= .07) and Constipation (F_2,49 _= .31; Eta^2 ^= .01) were not statistically significant at p < .05.

**Figure 3:**
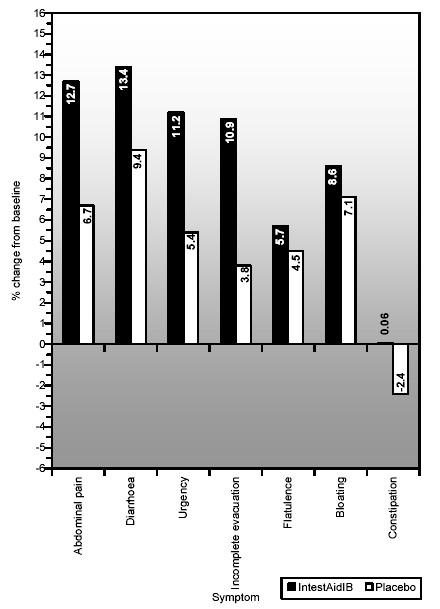
bar chart showing percentage change from baseline to placebo and experimental conditions.

The percentage improvement of both placebo and IntestAidIB over baseline is shown below (see Figure 3).

Symptom severity for all symptoms (except constipation) are in the expected direction of baseline>placebo>IntestAidIB.

### Psychological measures

Repeated measures ANOVA were carried out on the psychological variables. There were no significant differences between conditions on these measures (p > .05).

It might be expected that due to the benefits of being in a clinical trial, anxiety and depression would decrease over time. We thus tested this by a repeated measures analysis for CES-D and Health Anxiety and Stait-Trait Anxiety comparing measures at baseline, washout and end of trial without considering condition (placebo or experimental). None of these measures were statistically significant (all p > .05)

## Discussion

The study has shown that there is a consistent improvement in most of the symptoms of irritable bowel syndrome following nucleotide supplementation with IntestAidIB. There was a very low drop-out rate (18%); none of the participants who completed the study reported any side-effects. A feeling of urgency to have a bowel movement and abdominal pain showed the most improvement over baseline and placebo, and since abdominal pain is the symptom most likely to prompt people to seek medical help, this is a important finding. A feeling of incomplete evacuation after a bowel movement also improved following treatment by the neutroceutical product. However, whilst statistically significant, improvements are modest, and this may be due to several factors. Firstly, people with IBS are not a homogenous group, and show great variability in frequency and severity of symptoms, both from each other, and individually. This makes it difficult to detect effects of interventions. Secondly, the placebo response was strong – Spiller [16] states that it takes approximately 12 weeks for the placebo effect to reduce. Perhaps a longer trial – and with a larger dose – effects might have been stronger. However, this is speculative and further trials need to take these factors into consideration.

Participants were a mixed group of people who have a current diagnosis of IBS, some of whom still attend a gastroenterological clinic, and some of whom do not. They were not obtained from tertiary centres and carried on with their normal life taking their usual medications. Although their symptoms were not perhaps as severe as tertiary patients, their symptoms were bothersome enough for them to enter the clinical trial, and the symptoms were severe enough for the participants to buy a range of products aimed at relieving their symptoms. We expect that the effects found in this simple would be stronger if replicated with participants from tertiary centres.

The benefits over placebo compare favourably with benefits found in some drug trials. For example, Tegaserod (a drug which acts as a selective agonist at 5HT receptors in the gastrointestinal tract) was found to produce 4.7% advantage over placebo in the participants' assessment of global relief of IBS [24]. Although many such drugs are well tolerated, there were no side effects at all reported in the present study, which is a considerable advantage. The percentage improvement varied according to the symptoms – between 4 and 6%. The symptom improvements shown are unlikely to be the result of a decrease in anxiety or depression which has sometimes been cited as a reason for any improvement in trials, as anxiety and depression in the present study did not decrease significantly as the trial progressed (irrespective of condition). The effect – at least at this dosage – does not seem strong enough to lead to an improvement in psychological state since depression, anxiety, general health and illness intrusiveness did not differ between the conditions.

The strong placebo effects found in the present study are similar to those in other IBS- studies [16]. We suggest that the percentage improvement over and above the placebo effect is a physiological effect of the nucleotide supplement on the gut. However, whilst results were consistent and some symptoms were statistically significant, these effects may not be strong enough to be perceived as a great improvement by the participants, who will try to assess the benefits of such a supplement against a background of extremely variable symptoms. The mechanism by which nucleotide supplementation might improve gut function could be via increased mucosal protein, DNA and villus height – as has been found in animal studies [14,25] Evans et al [14] state that rodent models are useful in translational research to identify potential new treatments to increase gut mucosal growth that is potentially relevant to humans with short bowel syndrome (this is not related to IBS). They state "...an increase in the surface area that would correlate with the increased villus height and crypt depth may conceivably correspond to an increase in available nutrient transporters, which may translate into increased nutrient uptake" (Although people with IBS have not been shown to have damage to the gut, nucleotide supplementation may improve gut function nonetheless by such a mechanism. However, this is purely speculative, and the present study set out only to determine whether symptoms of IBS were improved with nucleotide supplementation. Further studies, preferably dose-dependent studies, will need to be carried out to determine the mechanism by which any improvements occur.

## Conclusion

A neutroceutical product, IntestAidIB, was found to improve six of the measured seven symptoms of IBS compared to both baseline and placebo. However, only abdominal pain and urgency to have a bowel movement showed statistically significant effects at the p < .05 level. Although the improvements in symptoms were consistent, the effects were not strong, and psychological measures showed no improvement either as a result of the experimental condition, or due to the benefits of taking part of a clinical trial. Further studies need to replicate and extend these results, seeking to clarify the mechanism by which improvements occur.

## Competing interests

This one-year study was funded by Wyreside Products Ltd, who provided us with information relating to the product and nucleotides, and the product itself. Wyreside Products Ltd were not involved in the design, running, analysis or write up of the study.

CPD and EA designed the study; KFB was employed as a research assistant and was in charge of the day-to-day running of the trial. All contributed to entering the data, data analyses, and writing the article.
